# Characterizing Occupational Complexity: Insights from the Canadian Longitudinal Study on Aging

**DOI:** 10.1093/geroni/igaf122.2899

**Published:** 2025-12-31

**Authors:** Catherine Gosselin, Annick Parent-Lamarche, Benjamin Boller

**Affiliations:** Université du Québec à Trois-Rivières, Repentigny, Quebec, Canada; Université du Québec à Trois-Rivières, Trois-Rivieres, Quebec, Canada; Université du Québec à Trois-Rivières, Trois-Rivières, Quebec, Canada

## Abstract

Using data from the Canadian Longitudinal Study on Aging (CLSA), we found that previous research suggests cognitive trajectories following retirement exhibit heterogeneity (Gosselin & Boller, 2022). One potential explanatory factor is the complexity of one’s profession, which has been linked to cognitive reserve theory. However, the specific structure of occupational complexity within this cohort remains to be fully characterized. This study aims to identify and define the key dimensions of occupational complexity among participants from the CLSA using a data-driven approach. The sample consisted of 8,243 workers (M = 54.18, SD = 6.09). Occupational information was classified using the Dictionary of Occupational Titles (DOT) to derive complexity scores based on levels of interaction with data, people, and materials. A principal component analysis (PCA) was conducted to extract underlying dimensions of occupational complexity. PCA revealed two distinct dimensions of occupational complexity, explaining 82.5% of the variance: the first, “Coordination and Support” (51.4%), encompasses professions characterized by high levels of social interaction, mentoring, and personnel management (e.g., psychologists). The second, “Autonomous Management” (31.1%), comprises professions requiring elevated autonomy in decision-making and the performance of specialized, often technical, tasks (e.g., engineers). These dimensions highlight distinct patterns of professional task demands within the CLSA cohort. By identifying key occupational complexity profiles, this study enhances our understanding of the professional backgrounds within the CLSA. These findings provide a foundation for future research examining the potential long-term cognitive and health-related implications of occupational characteristics.

